# Sexual Differences in Addictions and Dual Disorders: Importance of Gender Perspective

**DOI:** 10.3390/brainsci12101346

**Published:** 2022-10-04

**Authors:** Ana Benito, Fernando Rodríguez de Fonseca, Gonzalo Haro

**Affiliations:** 1 TXP Investigation Group, Medicine Department, Universidad Cardenal Herrera-CEU, CEU Universities, 12006 Castellón, Spain; 2 Torrente Mental Health Unit, General University Hospital, 46900 Valencia, Spain; 3 Institute of Biomedical Research of Malaga, 29010 Malaga, Spain; 4 Department of Psychobiology, Complutense University of Madrid, 28040 Madrid, Spain; 5 Department of Mental Health, Consorcio Hospitalario Provincial de Castellón, 12002 Castellon de la Plana, Spain

Sex (the biological attributes of females and males) and gender (socially constructed roles, behaviours, and identities) both affect molecular and cellular processes, protective and vulnerability factors, social and relational life [1], clinical features, seeking and responding to treatments, and health and illness [2]. Basic, preclinical, and clinical research has shown the presence of biological differences between the sexes from the beginning of embryonic development and throughout the entire life cycle [1]. Brain-imaging methods show sex-related differences in brain size, global and regional grey matter volume, white matter connectivity, and neuroanatomical regulation of appetite and satiety [3]. Sex differences have also been found in terms of the aetiology, prevalence, progression, severity, treatment efficacy, risk of side effects [4], and outcomes of multiple human diseases, including psychological, autoimmune, gastrointestinal, cardiovascular, renal, and reproductive illnesses [3]. In addition, males and females metabolise drugs differently, with the latter presenting more adverse reactions with more serious effects [4].

Despite all these data indicating that biological sex is a fundamental source of intraspecific variation in anatomy and physiology, most basic and clinical science has tended to focus only on one sex (typically males) [3]. Moreover, there is also a sex bias in both in vivo and in vitro preclinical research, which tends to focus mainly on male animals [4]. However, in 2014, the National Institute of Health (NIH) highlighted the need to study males and females in animal, tissue, and cell studies, and in 2015, it urged that sex be included as a biological variable in the research designs, analyses, and reporting of vertebrate animal and human studies [5]. This meant collecting data from both sexes to analyse sex as a source of potential variation and visualising and summarising this data by sex [4]. Given that sex differences permeate biology and medicine, they must also be considered in all areas of biomedical research [3].

Despite these recommendations, sex differences are poorly studied within the field of mental health, even though there is evidence of disparities (with respect to brain anatomy, activation patterns, and neurochemistry, etc.) that can significantly influence the aetiology and course of mental disorders [6]. The same occurs in the study of addictions and dual diagnosis, where research continues to be carried out mainly in male patients [1]. Therefore, this Special Issue aims to update our knowledge of the sexual and gender differences found in research into addictions and dual diagnosis. Thus, the articles included in this special issue cover both issues related to gender [1,7] and sex [6,8,9], patients using various substances [1,6,7] or others with specific substance use disorders (SUDs) [8,9], and including both issues related to addictions [9] and dual diagnosis [1,6,7,8].

Torrens-Melich et al. (2021) [1] highlighted the importance of the gender perspective in the study of dual diagnosis. They pointed out that there is little knowledge of the specific characteristics and needs of women with dual diagnosis, which contributes to the androcentric design of interventions, resources, and treatment services. They concluded their article by highlighting the need for a transversal sex- and gender-based perspective to properly study and treat dual diagnosis. In this sense, they argue that (1) factors related to sex and gender must be included in all analyses; (2) professionals must review their prejudices and stereotypes and train from a gender perspective; (3) administrations should design and provide specific treatment resources for women; and (4) that we can all contribute to a structural transformation of society that transcends gender mandates and norms and reduces the risk of abuse and violence against women.

In turn, Prieto-Arenas et al. (2022) [8] reviewed the literature on sex differences in the development of psychotic, depressive, and anxious symptoms associated with cannabis use. The authors also reviewed the possible causes of these differences. They concluded by emphasising that, although these results are inconclusive, they seem to indicate a greater vulnerability of women to the development of psychosis and anxiety with long-term cannabis misuse, with men being more vulnerable to the development of depressive symptoms. They also pointed out some possible explanations for the causes of these differences, including (1) the role of oestrogens and its relationship with the hypothalamic–pituitary–adrenal axis for the sex differences in the neuroendocrine stress response; (2) sex differences in the endocannabinoid system; and (3) the role of this neurotransmission system in the development of the brain and in the function of other important neurotransmission systems such as the dopaminergic, glutamatergic, and GABAergic systems.

For their part, Flores-López et al. (2002) [9] conducted an exploratory study to examine the plasma concentrations of lysophosphatidic acid (LPA, 1-acyl-2-hydroxy-sn-glycero-3-phosphate) species in women and men from a cohort of abstinent patients diagnosed with lifetime alcohol use disorder (AUD) and/or cocaine use disorder (CUD). The main results of their study were: (1) there are sex differences in LPA concentrations, which were higher in women; (2) there was a positive correlation between 16:0-LPA, 18:1-LPA, 18:2-LPA, 20:4-LPA, or total LPA concentrations and age in the SUD and control groups; (3) patients with SUD displayed lower 16:0-LPA, 18:2-LPA, and total LPA concentrations than healthy controls; (4) 16:0-LPA, 18:2-LPA, and total LPA concentrations were affected both by the diagnosis of a SUD and by sex, although there were no interactions between these two factors; and (5) the different species of LPA and total LPA concentrations were affected by the type of SUD: patients with CUD and AUD + CUD had lower LPA concentrations than the control or AUD groups. The authors concluded that LPA species were altered in patients with SUD compared to healthy controls and that there was sexual dimorphism in the plasma LPA concentrations in both control and SUD groups. Thus, they believe further exploration of the role of LPA as a potential biomarker for SUD may contribute to better patient stratification in treatment programs.

Castellano-Garcia et al. (2022) [6] reviewed sex differences in the prevalence of SUDs, pharmacological therapy, and mental health in adolescents (aged 13–18 years) diagnosed with attention deficit hyperactivity disorder (ADHD). The main conclusions of their study were that (1) girls with ADHD were more at risk of substance use than boys, although there was no consensus on the prevalence of dual disorders; (2) the prevalence of ADHD was lower in girls: fewer girls were diagnosed with ADHD than boys and they had fewer symptoms and so were less likely to receive treatment; (3) girls with ADHD scored higher for cognitive and motor impulsivity and had more impaired neuropsychological profiles in terms of executive functions, although ADHD was associated with worse academic performance in both sexes; (4) ADHD was related to psychological problems and distress in both sexes, although girls may have a greater tendency towards self-injurious behaviour; and (5) early diagnosis and treatment of ADHD, especially in adolescent girls, is essential to prevent early substance use, the development of SUDs, and suicidal behaviour.

Finally, Cotaina et al. (2022) [7] conducted a meta-analysis to group and analyse previously published results in order to quantify the probability (pooled odds ratio [OR]) of transgender people presenting with substance use and SUD compared to the cisgender population. Their results indicated that transgender individuals have a greater probability of current tobacco use (OR = 1.65; 95% CI [1.37, 1.98]) and use of specific substances (OR = 1.79; 95% CI [1.54, 2.07]), and of having consumed any substance over their lifetimes (OR = 1.48; 95% CI [1.30, 1.68]). However, when current alcohol and substance use in general, and tobacco, alcohol, and SUDs were specifically considered, the likelihood did not differ from that of cisgender people. The authors reached the conclusion that (1) although transgender participants were more likely to use tobacco and substances, this probability was not much higher than that of cisgender individuals; (2) consumption in transgender people can be an emotional regulation strategy, a maladaptive mechanism for coping with traumatic experiences, or could respond to minority stress produced by stigma, prejudice, discrimination, and harassment; (3) there were no differences for alcohol use or for any SUDs, and so considering this population as consumers or as addicted may be a prejudice that perpetuates stigma; (4) it is of particular importance to implement policies against discrimination and stigmatisation and to adapt prevention and treatment services so that they are inclusive of the 2SLGBTQIA+ community.

In summary, the articles included in this Special Issue show the relevance of sexual and gender differences in people with addictions and dual diagnosis. However, showing that these differences exist is only the beginning; the next necessary step will be to investigate the mechanisms at the base of these differences in order to transfer this knowledge into clinical practice [10]. The relative contributions of sex hormones, genes, and the environment, as well as the observer effect must all be considered to understand disease mechanisms and sex differences in the protection against or exacerbation of disease [3]. Recognising and understanding these differences is essential to be able to move towards individualised precision medicine [4], where a sex- and gender-informed perspective would increase rigor, promote discovery, increase the relevance of biomedical research, and improve patient care [2].
